# Helical intensity-modulated Radiotherapy of the Pelvic Lymph Nodes with Integrated Boost to the Prostate Bed - Initial Results of the PLATIN 3 Trial

**DOI:** 10.1186/1471-2407-14-20

**Published:** 2014-01-14

**Authors:** Sonja Katayama, Gregor Habl, Kerstin Kessel, Lutz Edler, Juergen Debus, Klaus Herfarth, Florian Sterzing

**Affiliations:** 1 Department of Radiation Oncology, University Hospital Heidelberg, Im Neuenheimer Feld 400, 69120 Heidelberg, Germany; 2 Department of Biostatistics, German Cancer Research Center, Heidelberg, Germany; 3 Clinical Cooperation Unit Radiation Oncology, German Cancer Research Center, Heidelberg, Germany

**Keywords:** Prostate, Postoperative Radiotherapy, Antihormonal treatment, Pelvic lymph nodes, IMRT, Tomotherapy

## Abstract

**Background:**

Adjuvant and salvage radiotherapy of the prostate bed are established treatment options for prostate cancer. While the benefit of an additional radiotherapy of the pelvic lymph nodes is still under debate, the PLATIN 3 prospective phase II clinical trial was initiated to substantiate toxicity data on postoperative IMRT of the pelvic lymph nodes and the prostate bed.

**Methods:**

From 2009 to 2011, 40 patients with high-risk prostate cancer after prostatectomy with pT3 R0/1 M0 or pT2 R1 M0 or a PSA recurrence and either > 20% risk of lymph node involvement and inadequate lymphadenectomy or pN + were enrolled. Patients received two months of antihormonal treatment (AT) before radiotherapy. AT continuation was mandatory during radiotherapy and was recommended for another two years. IMRT of the pelvic lymph nodes (51.0 Gy) with a simultaneous integrated boost to the prostate bed (68.0 Gy) was performed in 34 fractions. PSA level, prostate-related symptoms and quality of life were assessed at regular intervals for 24 months.

**Results:**

Of the 40 patients enrolled, 39 finished treatment as planned. Overall acute toxicity rates were low and no acute grade 3/4 toxicity occurred. Only 22.5% of patients experienced acute grade 2 gastrointestinal (GI) and genitourinary (GU) toxicity. During follow-up, 10.0% late grade 2 GI and 5.0% late grade 2 GU toxicity occurred, and one patient developed late grade 3 proctitis and enteritis. After a median observation time of 24 months the PLATIN 3 trial has shown in 97.5% of all patients sufficient safety and thus met its prospectively defined aims. After a median of 24 months, 34/38 patients were free of a PSA recurrence.

**Conclusions:**

Postoperative whole-pelvis IMRT with an integrated boost to the prostate bed can be performed safely and without excessive toxicity.

**Trial registration:**

Trial Numbers: ARO 2009–05, ClinicalTrials.gov: NCT01903408.

## Background

Postoperative radiotherapy of the prostate bed (PBRT) is a standard procedure for patients with an increased risk of local recurrence. Three prospective randomised trials demonstrated that PBRT significantly increases the biochemical recurrence free survival (BFS) compared to observation: EORTC 22911 [1], SWOG 8794 [2] and ARO 96–02 [3]. Whether patients benefit from the inclusion of the pelvic lymph nodes in the radiooncological treatment volume (whole pelvis radiotherapy, WPRT) is still subject to debate even in the setting of definitive radiotherapy [4,5]. In the adjuvant setting, data on radiotherapy of the pelvic lymph nodes is even more limited. Spiotto et al. [6] performed a retrospective analysis of 160 patients after prostatectomy who obtained either irradiation of the prostate bed or WPRT as adjuvant or salvage radiotherapy. Of these, 114 patients were at high risk of lymph node involvement (Gleason score ≥ 8, initial PSA > 20 ng/ml, ECE, SVI or pN+) and received either WPRT (n = 72) or treatment of the prostate bed (n = 42) with or without a short course of neoadjuvant and concurrent total androgen suppression. Five-year-BFS was significantly higher in the WPRT group compared to prostate bed irradiation. A benefit could only be shown for high risk patients (5 year BFS 47% vs. 21%, for WPRT vs. PBRT, respectively). Likewise, Briganti et al. showed in a matched-pair analysis of 703 pT2-4 pN + patients that the combination of androgen deprivation and radiotherapy prolonged cause-specific and overall survival compared to androgen deprivation alone [7]. In contrast, a retrospective cohort study of 247 patients comparing WPRT vs. PBRT that excluded patients under androgen suppression only demonstrated a benefit regarding biochemical control for patients with a pretreatment PSA ≥ 0.4 ng/ml [8].

The PLATIN (Prostate and Lymph Node Irradiation with Integrated-Boost-IMRT after Neoadjuvant Antihormonal Treatment) phase II trial was initiated in 2009 to investigate safety and feasibility of an irradiation of the pelvic lymph nodes simultaneously with an integrated boost to either the prostate (PLATIN 1), the prostate and macroscopic nodes (PLATIN 2), the prostate bed (PLATIN 3), the prostate bed and macroscopic nodes (PLATIN 4) or to macroscopic nodes in patients that had received PBRT before (PLATIN 5). Secondary objectives were a detailed characterisation of the toxicity profiles of the respective treatments and the evaluation of quality of life.

This article reports first safety and efficacy data of the intensity-modulated radiotherapy (IMRT) treatment of the pelvic lymph nodes with a simultaneous boost to the prostate bed (PLATIN 3).

## Methods

Before trial initiation, ethical consent was obtained from the ethics committee of the University of Heidelberg, Germany (Medical Faculty). All patients gave written informed consent before inclusion in the trial.

From May 2009 to May 2011, 40 patients were enrolled prospectively in the PLATIN 3 trial. Eligibility criteria were a resected prostate carcinoma with pT3 R0/1 or pT2 R1 or with a postoperative PSA recurrence (defined as three consecutive PSA rises above the nadir). Additionally, pN + disease or an estimated risk of lymph node involvement > 20% according to the Roach formula [9] with inadequate nodal dissection (<10) were required.

Patients received at least two months of neoadjuvant AT which was continued during radiotherapy in all cases. Continuation was recommended for two years after irradiation.

For treatment planning, CT scans with 3 mm slice thickness at full bladder and empty rectum were performed. PTV-P (planning target volume - prostate bed) comprised the prostate bed including the bottom of the bladder and the anterior rectal wall with a margin of 0.5 cm. PTV-L (planning target volume - lymph nodes) included the obturatory, internal and external iliac, common iliac and presacral (down to S3) nodes [10] with a 0.5 mm margin. Pararectal nodes were not included in the PTV-L. Inverse treatment planning was performed using the Tomotherapy® Treatment Planning Software. A total dose of 51.0 Gy was prescribed to 95% of PTV-L with a simultaneous integrated boost of 68.0 Gy to 95% of PTV-P in 34 fractions. The dose prescription to the lymph nodes of 51 Gy in 1.5 Gy fractions is biologically equivalent to 43.7 Gy, assuming an α/β of 1.5 Gy for prostate cancer, and 48.2 Gy, assuming an α/β of 7 Gy for small bowel. Treatment with Helical Tomotherapy was performed with full bladder and empty rectum under daily image guidance.

Prostate-specific symptoms and treatment toxicity, using the criteria of the NCI CTC AE version 3.0, were recorded before treatment, weekly during treatment, at the end of treatment, and at 2.5, 6, 12, 18 and 24 months after start of treatment. For calculation of toxicity rates, only the patients with available data at the respective time points were considered. Cumulative GI toxicity was defined as the cumulative incidence of diarrhea, enteritis and proctitis. To facilitate comparison with other publications, only cystitis was included in the calculation of cumulative genitourinary toxicity, as most scoring systems do not include incontinence and erectile dysfunction. Nevertheless, incontinence and erectile dysfunction were recorded (according to NCI CTC AE version 3.0). Cumulative toxicity rates were calculated considering all 40 patients enrolled in the trial.

Quality of life was assessed using the EORTC QLQ-C30 questionnaire before and at the end of treatment and at 6, 12 and 24 months.

PSA levels were measured before the start of treatment and then every three months, starting from week 10. Biochemical failure was established when at three subsequent time points with a minimum interval of 4 weeks PSA levels increased continuously from the lowest measured PSA.

As primary endpoint, the safe treatment application rate (STR) was chosen. STR was defined as the proportion of patients receiving treatment as planned and without grade 3–4 toxicity and calculated as the ratio of the number of patients fulfilling this criteria divided by the size of the intent-to-treat (ITT) population. The ITT population consisted of all patients giving informed consent, fulfilling the inclusion/exclusion criteria and receiving planned treatment for a minimum of 4 weeks after initiation. Based on a one-stage phase II type design, STR of 80% (null-hypothesis STR ≤ 80%) was tested against the alternative of being at least as large as 95% in a one-stage phase-II type design using the exact Binomial test at the significance level of 0.1% with a power of 90%. The null hypothesis would be rejected when STR would be at least 87.7%.

## Results

### Patient characteristics

Among the 40 eligible patients (identical with the ITT population), median follow-up was 24 months (range: 15–24 months). Median age at inclusion was 68 years (range: 46–75 years); all patients had high-risk disease according to the D‘Amico risk categories [11] based on preoperative staging. The majority of patients (50%) had Gleason 9 tumors, while 20% and 30% were staged as Gleason 8 and 7, respectively. There were 23 patients with pN + disease and 17 patients with an estimated risk of lymph node involvement > 20% and inadequate lymphadenectomy. Radiotherapy was performed as adjuvant treatment in 30 patients and 10 patients received salvage radiotherapy for a PSA recurrence. Median time between prostatectomy and radiotherapy was 7 months for adjuvant treatment (range 2–17 months) and 14 months for salvage radiotherapy (range 7–56 months). Median PSA before the start of AT and radiotherapy was 0.4 ng/ml in the adjuvant setting and 0.19 ng/ml in patients receiving salvage radiotherapy.

Treatment was fully completed by 39/40 patiens. One patient developed diarrhea which was objectively scored as grade 2. However, the patient could not cope with the symptoms, refused supportive medication and further consultation and eventually refused treatment continuation after 22 fractions. Another patient developed low blood sodium during radiotherapy, was subsequently diagnosed with metastasised small lung cancer after finishing radiotherapy and was lost to follow-up shortly thereafter.

### Treatment characteristics

Average beam on time was 7:59 min (± 1:00 min). The intended target coverage could be met, as 95% of the PTV-P received 68.0 ± 0.67 Gy (median dose: 70.12 ± 0.62 Gy) and 95% of the PTV-L received 50.27 ± 0.43 Gy (median dose: 52.97 ± 0.73 Gy).

Plan quality in terms of organ at risk sparing is shown in Table 1. On average, the anterior rectal wall received a maximum dose of 71.4 Gy (range: 67.0 Gy - 71.9 Gy). Only small volumes of the rectum received doses ≥ 60 Gy (9.5%) and ≥ 70 Gy (1.0%), respectively. Dose to the small bowel could be kept at low levels with 10.3% of the small bowel exposed to ≥ 40 Gy and a maximum dose of 53.1 Gy. Most of the bladder could be spared from high dose exposure, with 3.5% of the bladder receiving ≥ 70 Gy.

**Table 1:** Average dose exposure to the rectum, small bowel and bladder

**Rectum**	
Maximum anterior rectal wall	71.4 Gy ± 1.1 Gy
V_40 Gy_	41.0% ± 13.8% / 41 ml ± 14 ml
V_60 Gy_	9.5% ± 5.7% / 10 ml ± 6 ml
V_70 Gy_	1.0% ± 1.5% / 1 ml ± 1 ml
**Small bowel**	
Maximum	53.1 Gy ± 1.2 Gy
V_20 Gy_	42.1% ± 15.2% / 672 ml ± 243 ml
V_40 Gy_	10.3% ± 6.3% / 164 ml ± 101 ml
**Bladder**	
V_40 Gy_	40.9% ± 8.8% / 125 ml ± 27 ml
V_60 Gy_	13.2% ± 8.2% / 40 ml ± 25 ml
V_70 Gy_	3.5% ± 3.6% / 11 ml ± 11 ml

### Treatment safety

After a median observation time of 24 months one patient terminated treatment prematurely but no patient showed acute toxicity ≥ grade 3. Therefore at this stage the PLATIN 3 trial has shown a promising STR of 97.5% (39/40) and has met the prospectively defined statistical criterion of a successful treatment with an STR of at least 87.7%.

### Gastrointestinal toxicity

Cumulative incidence of acute gastrointestinal (GI) toxicity was 67.5% (grade 1) and 22.5% (grade 2), respectively, but no acute grade 3/4 GI toxicity occurred. Investigation of different GI symptoms showed that at the end of treatment, the rates of grade 1 (< 4 stools increase over baseline) and 2 (4–6 stools increase over baseline) acute diarrhoea were 28.2% and 2.6%, respectively, while 17.9% of patients reported rectal discomfort not requiring intervention (proctitis grade 1) and 12.8% had symptoms of rectal discomfort or passing of mucus or blood that required medical intervention (grade 2). Enteritis grade 1 (asymptomatic, not requiring intervention) occurred in 15.4%.

Cumulative late GI toxicity was 7.5% (grade 1), 10.0% (grade 2) and 5.0% (grade 3), respectively. No patient suffered from late diarrhea of any grade, and only one patient experienced late proctitis and enteritis grade 3 at 18 months of follow-up (see Table 2).

**Table 2:** Acute and late gastrointestinal toxicity

**Diarrhea**	**Grade 1**	**Grade 2**	**Grade 3**	**Grade 4**
End of RT	28.2%	2.6%	-	-
13 weeks	7.9%	-	-	-
6 months	-	2.7%	-	-
12 months	2.7%	-	-	-
18 months	-	-	-	-
24 months	-	-	-	-
**Enteritis**	**Grade 1**	**Grade 2**	**Grade 3**	**Grade 4**
End of RT	15.4%	-	-	-
13 weeks	-	7.9%	-	-
6 months	-	5.4%	-	-
12 months	-	-	-	-
18 months	31.%	-	-	-
24 months	-	4.5%	-	-
**Proctitis**	**Grade 1**	**Grade 2**	**Grade 3**	**Grade 4**
End of RT	17.9%	12.8%	-	-
13 weeks	10.5%	-	-	-
6 months	2.7%	2.7%	-	-
12 months	-	-	-	-
18 months	-	-	3.1%	-
24 months	-	-	-	-

Diarrhea: CTC AE grade 1 = increase < 4 stools per day over baseline; grade 2 = increase 4-6 stools per day; grade 3 = increase ≥ 7 stools per day or incontinence or hospitalisation or limiting self care ADL; grade 4 = life-threatening consequences, urgent intervention indicated.

Enteritis: CTC AE grade 1 = asymptomatic; grade 2 = abdominal pain, mucus or blood in stool; grade 3 = severe or persistent abdominal pain, fever, ileus, peritoneal signs; grade 4 = life-threatening consequences, urgent intervention indicated.

Proctitis: CTC AE grade 1 = rectal discomfort, intervention not indicated; grade 2 = symptoms (e.g. rectal discomfort, passing blood or mucus), medical intervention, limiting instrumental ADL; grade 3 = severe symptoms, fecal urgency or stool incontinence, limiting self care ADL; grade 4 = life-threatening consequences, urgent intervention indicated.

### Genitourinary toxicity

Genitourinary (GU) toxicity was low with a cumulative incidence of acute GU toxicity grade 1 and 2 in 22.5% of patients each. Cumulative incidence of late GU toxicity was 22.5% (grade 1) and 5.0% (grade 2), respectively. No patient developed acute or late cystitis grade 3/4. At the end of treatment, mild acute cystitis was reported by 25.6% (grade 1) and 20.5% (grade 2) (see Table 3).

**Table 3:** Acute and late cystitis

**Cystitis**	**Grade 1**	**Grade 2**	**Grade 3**	**Grade 4**
End of RT	25.6%	20.5%	-	-
13 weeks	2.6%	7.9%	-	-
6 months	5.4%	8.1%	-	-
12 months	2.7%	2.7%	-	-
18 months	16.1%	-	-	-
24 months	-	4.5%	-	-

Cystitis: CTC AE grade 1 = asymptomatic; grade 2 = frequency with dysuria, macroscopic hematuria; grade 3 = transfusion, IV pain medications, bladder irrigation indicated; grade 4 = catastrophic bleeding, major non-elective intervention indicated.

A substantial proportion of patients (62.5%) experienced urinary stress incontinence before the start of radiotherapy: grade 1 stress incontinence (occasional, no pads necessary) was present in 47.5% of patients, 15.0% had spontaneous loss of urine and needed pads (grade 2). Severity of stress incontince varied during follow-up: Twelve months after treatment, 35.1% reported grade 1 and 29.7% grade 2 incontinence, respectively. After 24 months, grade 1 stress incontinence was present in 14.3% of patients and grade 2 in 19.1% of patients.

Urge incontinence increased after treatment from 12.5% and 2.5% of patients suffering from grade 1 and 2 incontinence before treatment to 21.6% and 5.4% after 12 months and 14.3% and 4.8% after 24 months, respectively.

Two patients required urinary catheterisation during treatment and one during follow-up due to urinary retention. At 24 months of follow-up, all patients that had completed follow-up were catheter-free.

Directly after surgery and even before the start of AT, a large proportion of patients (85%) reported a complete loss of erectile function. This percentage increased during the course of treatment and follow-up to 95% (see Table 4). Before the start of AT, 21% of patients had experienced no change in libido while 28.2% reported a complete loss of libido. The latter proportion increased with the duration of AT up to 77% after 24 months.

**Table 4:** Erectile function

**Erectile function**	**Grade 0**	**Grade 1**	**Grade 2**	**Grade 3**
Before AT	2.6%	7.7%	5.1%	84.6%
After 2 months of AT	-	2.6 %	5.1%	92.3%
End of RT	2.6%	2.6%	12.8%	82.1%
13 weeks	2.6%	7.9%	10.5%	78.9%
6 months	2.7%	5.4%	8.1%	83.8%
12 months	2.8%	2.8%	8.3%	86.1%
18 months	-	-	9.7%	90.3%
24 months	-	-	4.8%	95.2%

Erectile function: CTC AE grade 1 = erectile dysfunction; grade 2 = decrease in erectile function (frequency/rigidity of erections) but erectile aids not indicated; grade 2 = decrease in erectile function, but erectile aids not helpful.

### Quality of life

Overall health as assessed by the “Global Health Score” of the EORTC QLQ-C30 questionnaire remained almost unchanged at the three investigation times 6, 12 and 24 months compared to baseline with and average score of 67.1 before radiotherapy and 73.2 after 24 months. Scores were on a similar level as the EORTC reference value (68.4) of prostate cancer patients over all disease stages. Similarly, the other scores involving physical, emotional, cognitive and social functioning as well as role functioning were constant over the course of treatment and observation period (data not shown).

### Biochemical control and survival

During follow-up, four patients experienced PSA recurrences, two of which were under AT at that time. In one patient, PSA recurrence coincided with the diagnosis of bone metastases; in the other three patients, the site of recurrence could not be determined. At the time of analysis, 19 patients still received AT. Average duration of AT was 11.5 months for the 38 patients remaining in the trial after the end of radiotherapy and 37 were known alive at a median of 24 months after start of treatment. One patient had developed acute myeloid leucemia (AML) at 14 months of follow up and had eventually died of the disease.

## Discussion

While the role of adjuvant radiotherapy of the whole pelvis remains controversial, this prospective trial shows good tolerability of an IMRT-based treatment of the pelvic lymph nodes. At the point of analysis, 34 of 38 patients were free of biochemical recurrence. For a reliable evaluation of efficacy, however, a median follow-up of 24 months is not sufficient.

Helical IMRT of the pelvic lymph nodes with a simultaneous integrated boost to the prostate bed could be performed in satisfying speed with an average treatment time of 8 min.

In two patients, a secondary malignancy (small cell lung cancer and AML) was diagnosed during follow-up. Since both occurred within 12 months after radiotherapy, the probability for them being radiation-induced is low.

In the present trial, no patient developed acute or late grade 3/4 genitourinary toxicity or acute grade 4 gastrointestinal toxicity. Only one patient experienced late grade 3 proctitis and enteritis 18 months after the start of treatment. This patient had a history of resection of rectal cancer with a subsequent anal stenosis requiring multiple treatments 20 years prior to prostate cancer treatment. During follow-up, a peak (5/31 patients) of grade 1 cystitis occurred at 18 months, which had subsided in 2 of 3 patients at the next visit.

Although the evaluation of erectile function based on patient-reported data is prone to reporting bias, we could detect only a small effect of postoperative radiotherapy on erectile function. However, compared to literature, a large proportion of patients in our trial (85%) initially reported complete loss of erectile function, probably due to the fact that all patients had high-risk disease and consecutively extensive surgery.

One limitation of this trial is the follow-up of currently two years. Further observation time is needed for a final evaluation of late toxicity, since especially late GU toxicity can occur up to ten years after treatment. In this trial, no direct comparison to standard irradiation of the prostate bed was performed. For further evaluation of clinical efficacy, a randomised prospective trial with a far larger number of patients and a more homogeneous patient group is warranted.

In addition, since a conventional fractionation was intended for the prostate bed, the daily dose given to pelvic lymphatic drainage (1.5 Gy) was lower than conventional fractionation. This might have a negative effect on the efficacy of WPRT.

Comparison of our toxicity data to trials on postoperative radiotherapy of the prostate bed [1-3] is complicated by the fact that different scoring systems were used and the applied dose, the manner of toxicity reporting and the follow-up time vary between trials. Yet, our results compare favourably to the treatment of the prostate bed alone (see Tables 5 and 6): First of all, like in all three trials, we saw no grade 4 acute or late toxicity. The EORTC trial [1,12] reported a 5-year cumulative incidence of 4.2% grade 3 toxicity. In the ARO trial [3], the rate of grade 3 toxicity was 0.3%. With 1/40 patients with grade 3 enteritis and proctitis our results are concurring with that data. A more recent publication on postoperative prostate bed irradiation [13] also stated similar toxicity: Among 182 patients treated with a median dose of 66.6 Gy to the prostate bed with 3D-CRT, acute GU and GI toxicity occurred in 39.6% and 50% of patients, respectively. Only two patients experienced acute grade 3 GI toxicity. Late GU and GI toxicities were seen in 15.4% and 7.7%, with one grade 3 GI side effect and 10 GU toxicities.

**Table 5:** Publications on acute toxicity of radiotherapy of the prostate bed with or without pelvic irradiation

		**Acute grade 1**		**Acute grade 2**		**Acute grade 3**		**Acute grade 4**	
		**GI**	**GU**	**GI**	**GU**	**GI**	**GU**	**GI**	**GU**
Bellavita et al. [13]	3D-CRT prostate bed 66.6 Gy	48.9% (G1/2)	-	-	-	1.1%	-	0%	0%
**Current trial**	**IMRT WPRT 51 Gy + boost prostate bed 68 Gy**	**67.5%**	**22.5%**	**22.5%**	**22.5%**	**0%**	**0%**	**0%**	**0%**
**Alongi et al.**[14]	**IMRT WPRT 50.6 Gy + boost prostate bed 70–72.5 Gy**	**-**	**-**	**3.3%**	**6.6%**	**0%**	**0%**	**0%**	**0%**
**Deville et al.**[15]	**IMRT WPRT 45 Gy Gy + boost prostate bed 70.2 Gy**	**31%**	**67%**	**61%**	**22%**	**0%**	**0%**	**0%**	**0%**
*Alongi et al.*[14]	*3D-CRT WPRT 50.1 Gy + boost prostate bed 72.1 Gy*	*-*	*-*	*8.6*%	*12.3*%	*0*%	*0*%	*0*%	*0*%
*Aizer et al.*[16]	*3D-CRT WPRT 45 Gy + IMRT boost prostate 75.6 Gy*	*75*%	*44.1*%	*17.6*%	*33.8*%	*1.5*%	*10.3*%	*0*%	*0*%
*Roach et al.*[4]	*3D-CRT WPRT 45 Gy + boost prostate 70.2 Gy*	*-*	*-*	*-*	*-*	*2.6*% *(G3/4)*	*3.9*% *(G3/4)*	*0*%	*0*%
*Liu et al.*[17]	*2D WPRT 45 Gy + boost prostate 72 Gy*	*12.2*%	*10.9*%	*1.3*%	*3.8*%	*0.6*%	*0.6*%	*0*%	*-*

Published data on acute toxicity of adjuvant 3D conformal radiotherapy (3D-CRT) of the prostate bed (regular print), adjuvant intensity-modulated radiotherapy (IMRT) of the whole pelvis (WPRT) with a boost to the prostate bed (bold print) and definitive or adjuvant 3D-CRT WPRT with a boost to the prostate or prostate bed (italic print). (GI = gastrointestinal, GU = genitourinary).

**Table 6:** Publications on late toxicity of radiotherapy of the prostate bed with or without pelvic irradiation

		**Median follow-up**	**Late grade 1**		**Late grade 2**		**Late grade 3**		**Late grade 4**	
			**GI**	**GU**	**GI**	**GU**	**GI**	**GU**	**GI**	**GU**
Bolla et al. [1]	3D-CRT prostate bed 66.0 Gy	60 months	-	-	-	-	4.2%		0%	0%
Bolla et al. [12]	3D-CRT prostate bed 66.0 Gy	127 months	-	-	-	-	5.3%		0%	0%
Wiegel et al. [3]	3D-CRT prostate bed 66.60 Gy	54 months	-	-	1.4%	2.0%	-	0.5%	0%	0%
Bellavita et al. [13]	3D-CRT prostate bed 66.6 Gy	56 months	7.1% (G1/2)	9.9% (G1/2)	-	-	0.5%	5.5%	0%	0%
Ost et al. [18]	IMRT/3D-CRT prostate bed 69.1 Gy	60 months	∼ 28%	∼ 34%	∼ 5%	∼ 17%	< 1%	10%	0%	0%
**Current trial**	**IMRT WPRT 51 Gy + boost prostate bed 68 Gy**	**24 months**	**7.5%**	**22.5%**	**10.0%**	**5.0%**	**5.0%**	**0%**	**0%**	**0%**
**Deville et al.**[15]	**IMRT WPRT 45 Gy Gy + boost prostate bed 70.2 Gy**	**26 months**	**11%**	**50%**	**3%**	**17%**	**0%**	**11%**	**0%**	**0%**
*Aizer et al.*[16]	*3D-CRT WPRT 45 Gy + IMRT boost prostate 75.6 Gy*	*30 months*	*2.9*%	*1.5*%	*4.4*%	*1.5*%	*1.5*%	*0*%	*0*%	*0*%
*Roach et al.*[4]	*3D-CRT WPRT 45 Gy + boost prostate 70.2 Gy*	*-*	*-*	*-*	*-*	*-*	*4.3*% *(G3/4)*	*3.0*% *(G3/4)*	*-*	*-*

Published data on late toxicity of adjuvant 3D conformal radiotherapy (3D-CRT) of the prostate bed (regular print), adjuvant intensity-modulated radiotherapy (IMRT) of the whole pelvis (WPRT) with a boost to the prostate bed (bold print) and definitive or adjuvant 3D-CRT WPRT with a boost to the prostate or prostate bed (italic print). (GI = gastrointestinal, GU = genitourinary).

Although data on the benefit of WPRT is sparse, many radiation oncology centers electively treat the pelvic lymph nodes because of publications on surgical lymph node sampling and nanoparticle-enhanced MRI studies that revealed a high proportion of occult lymph node metastases [19,20]. In contrast, other centers avoid WPRT in because of concerns about excessive toxicity.

In recent years, first clinical trials [14,15] provided toxicity data on postoperative IMRT treatment of the pelvic lymph nodes (see Tables 5 and 6). Alongi et al. [14] showed for the first time that acute WPRT toxicity could be lowered substantially with the use of IMRT. Among 172 patients that received postoperative WPRT with either a 3D conformal technique or IMRT, acute ≥ grade 2 GU and GI toxicity was significantly lower in the IMRT group. In their series of 67 patients receiving either 45 Gy IMRT to the whole pelvis and a 70.2 Gy boost to the prostate bed or prostate bed irradiation alone, Deville et al. [15] described an increase in acute grade 2 GI toxicity in the WPRT group. However, acute ≥ grade 2 GU toxicity as well as late ≥ grade 2 GI or GU toxicity was not increased with WPRT.

The currently recruiting RTOG 0534 trial will provide randomized data on postoperative WPRT: patients with pT2-3 pN0/Nx R0/1 tumors Gleason ≤ 9 with a rising PSA (≥ 0.1 - < 2.0 ng/ml) after prostatectomy receive neoadjuvant and concomitant short term antihormonal treatment and either WPRT or PBRT.

Until results of the RTOG 0534 trial have matured, the role of adjuvant WPRT will remain under discussion. So far, however, the results of this trial and the previously published data confirm that WPRT for postoperative prostate cancer is well tolerated when state-of-the-art IMRT techniques are applied.

## Conclusions

While the role of pelvic irradiation in the postoperative treatment of prostate cancer remains to be fully explored, we could demonstrate in this prospective trial that prophylactic radiotherapy of the pelvic lymph nodes with an integrated boost to the prostate bed can be performed without excessive toxcitiy. Further prospective clinical trials should assess clincial efficacy in patients with high risk of pelvic lymph node metastases.

## Competing interests

The Department of Radiation Oncology, University Hospital Heidelberg, has a research collaboration with Accuray Inc., who is the manufacturer of Tomotherapy® machines and software.

## Authors‘ contributions

SK was performed patient treatment and follow up and data acquisition and drafted the manuscript. GH performed treatment and follow up. KK was responsible for data management. LE planned the trial statistics. JD revised the manuscript. KH was the principal investigator and revised the trial protocol and the manuscript. FS designed the trial protocol, was responsible for patient treatment and follow up and revised the manuscript. All authors read and approved the final manuscript.

## Pre-publication history

The pre-publication history for this paper can be accessed here:

http://www.biomedcentral.com/1471-2407/14/20/prepub
